# *Nocardia* and the Death of Raphael (1520)

**DOI:** 10.3201/eid3201.AC3201

**Published:** 2026-01

**Authors:** Philippe Charlier, Geneviève Héry-Arnaud, Kira Kofoed, Jean Armengaud

**Affiliations:** Laboratory Anthropology, Archaeology, Biology, UFR of Health Sciences Simone Veil, University of Versailles Saint-Quentin-en-Yvelines/ Université Paris-Saclay, Paris, France (P. Charlier); University Hospital R. Poincaré (AP-HP), Garches, France (P. Charlier); Brest University Hospital, Brest, France (G. Héry-Arnaud); Brest University, INSERM, EFS, UMR 1078 Inserm Unit UMR1078 Genetics, Genomics and Biotechnology, Faculty of Medicine, Brest (G. Héry-Arnaud); Thorvaldsens Museum, Copenhagen, Denmark (Kira Kofoed); Université Paris-Saclay, CEA, INRAE, Bagnols-sur-Cèze, France (J. Armengaud)

**Keywords:** Nocardia, bacteria, Raphael, art–science connection, paleopathology, forensic medicine, history of medicine, infection

**Figure 1:**
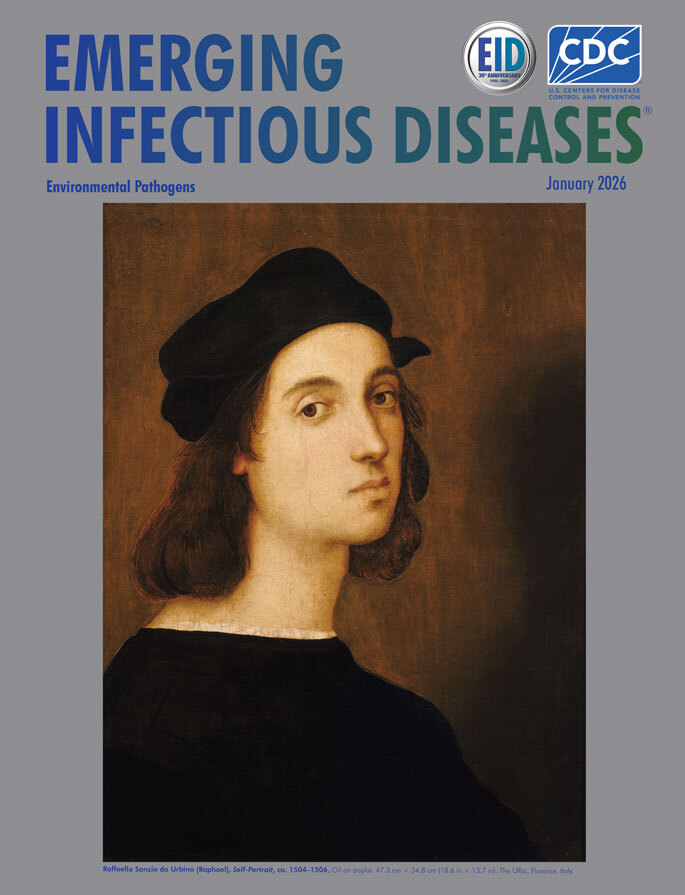
**Raffaello Sanzio da Urbino (Raphael), *Self-Portrait*, ca. 1504–1506.** Oil on poplar. 47.3 cm × 34.8 cm (18.6 in × 13.7 in). The Uffizi, Florence, Italy.

Raphael (Raffaello Sanzio da Urbino) was one of the greatest artists of the Italian Renaissance. Active in Perugia, Siena, Florence, Rome, and Urbino (his birthplace), he is responsible for numerous masterpieces of Western art, including the fresco of the School of Athens in the Vatican rooms and the painting of the Sistine Madonna accompanied by 2 cherubs (preserved in Dresden, Germany). He died at the age of 37 in Rome, Italy, on April 6, 1520, after a short chest infection with fever. Several hypotheses have been proposed to explain his premature death, including syphilis, typhoid, malaria, and even excessive bloodletting (*1*). The paleoproteomic study of his remains might be capable of revealing the exact cause of his death.

Raphael’s body was placed in one of the walls of the Pantheon in Rome, where his remains are entombed to this day. However, in 1833, an opening of the grave was carried out at the request of the Pope Gregory XVI to check the condition of the corpse and place it in a marble sarcophagus from the Vatican museums (Figure 1). On that occasion, casts of the skull and right hand were made, a piece of the heart was given to a Russian diplomat to the Holy See (*2*), and the Danish sculptor Bertel Thorvaldsen was given a fragment of the mortar hanging from the original coffin: he kept the fragment and later donated it to his Danish house servant (now at the Thorvaldsens Museum in Copenhagen, inv. no. N87). The French painter Jean-Auguste-Dominique Ingres, who was not present at the opening of the tomb, was given permission by the pope to have a piece of Raphael’s bones (*3*). Keeping bone fragments as relics had been a practice for hundreds of years, especially when associated with religious figures. Raphael was highly venerated as an artist, to such an extent that he was considered almost a saint (*2*,*3*).

**Figure 2:**
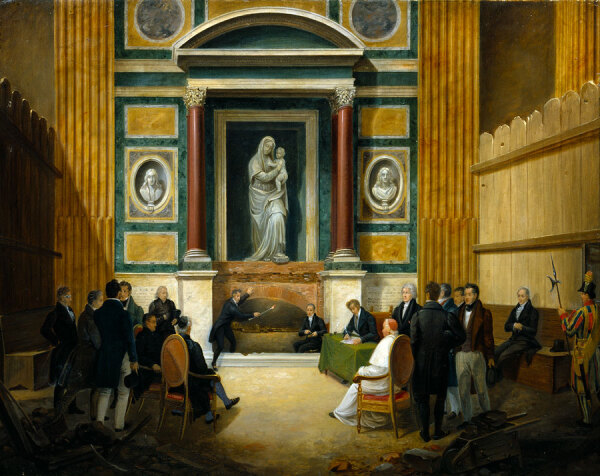
Francesco Diofebi, *The 1833 Opening of Raphael’s Tomb in Pantheon*, 1836. Oil on canvas, 54.9 × 70 cm, Thorvaldsens Museum inv. no. B73. Photograph by Hans Petersen.

In the archives of Dominique Ingres, in Montauban (France), we found envelopes and a glass frame holding what were described by Ingres himself as “remains of the divine Raphael” (bone splinters and sediments; Figure 2). Because of the small amount of remaining bone material, no carbon-dating of the remains has been conducted to possibly confirm their authenticity. Apart from tradition (not questioned by art historians) and archives relating to the relics belonging to the Thorvaldsesn Museum in Copenhagen (*4*), no conclusive evidence exists to confirm the authenticity of the Montauban’s remains.

**Figure 3:**
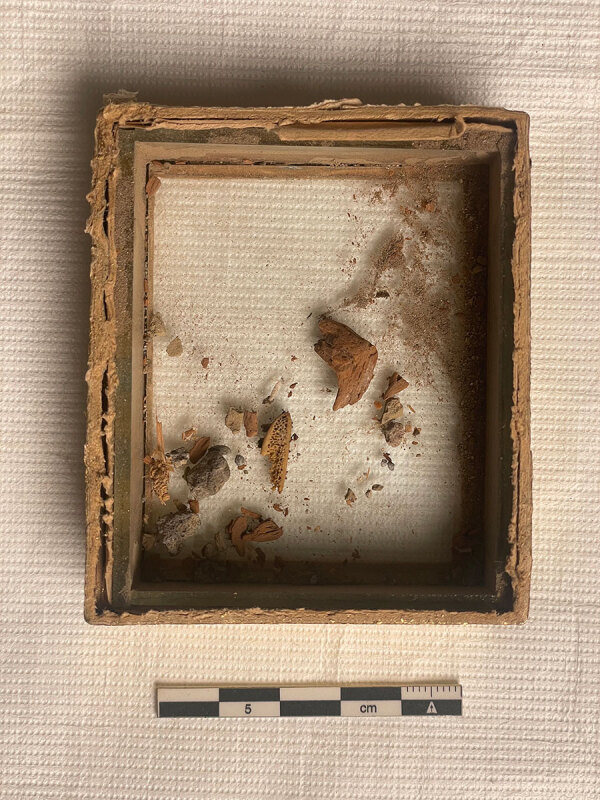
General view of samples from Raphael’s grave and cadaver analyzed for investigation of possible *Nocardia* infection. Ingres Museum, Montauban, France. Photograph by Philippe Charlier.

Nevertheless, the fragments were the subject of a sampling for a proteomic study, according to a now well-established paleopathological protocol (paleo-proteotyping) (*5*). The analysis revealed a significant proteomic signature for *Nocardia niigatensis* (50 peptides assigned, 11 of them species-specific peptides, and high redundancy of 91 spectra by tandem mass spectrometry), and the bacterium was detected only in the sample of bone marrow from a fragment of human rib. In contrast, several other bacteria, typically environmental, were detected in several of the other samples of the grave soil (*Pseudonocardia autotrophica*, *Nonomurae gerenzanensis*, *Streptomyces albireticuli*, *Hyphomicrobium* sp., *Paraburkholderia ribeironi*, *Lysobacter antibioticus*, *Blastocatella* sp., *Paenibacillus koleovorans*, *Arcicella rosea*, *Bacillus megaterium*, and *Rhizobium leguminosarum*). Finding *Nocardia* exclusively in the bone marrow supports a conclusion that *Nocardia* might have been a cause of death, especially since the dead body was placed in an above-ground niche within an ancient Roman monument that had been transformed into a church. No sign of embalming was found at the exhumation (*3*). 

*N. niigatensis* is not part of the thanatomicrobiome (i.e., microbial communities residing in or moving on the surface of altered remains), so its presence cannot be explained by a process of decomposition/putrefaction (*6*). However, one may consider the possibility of a burial contamination by the waters of the Tiber during the numerous floods that occurred from the 16th through the 19th Centuries (*7*). Indeed, the drawings (for example by Vincenzo Camuccini), paintings, and reports of the state of the skeleton during the exhumation of 1833 show a partially disarticulated skeleton, subsequently reassembled by the physician Antonio Trasmondo. The presence of slight deposits on Raphael’s bones, potentially resulting from Tiber River flooding, were described by some witnesses in 1833 (*8*). However, the peptide signature of *N. niigatensis* was found only in the bone marrow sample of the rib segment and not in any other samples (e.g., soil, wall, dust), suggesting an intravitam origin for this infectious agent, rather than postmortem contamination by secondary deposition. The detection of environmental bacteria, the presence of deposits on bones attributed to flooding, and difficulty confirming that the bone fragments and sediments were from Raphael and his tomb exemplify some of the challenges to draw conclusions based on a full paleopathological study.

*Nocardia* spp. bacteria can infect bones through direct extension from a primary lung or skin lesion or via hematogenous dissemination from a pulmonary or soft tissue focus. The clinical manifestations of a *Nocardia* infection typically begin as a low-level, indolent infection, which then can progress to a febrile illness characterized by pulmonary abscesses, disseminated infection, or both, including osteomyelitis. That clinical course aligns with the symptoms reported for Raphael’s last days of life and agony (*9*): we know from Vasari that Raphael’s death was preceded by high fever; another source speaks of a 15-day illness (letter from Alf. Pauluzzi to Duke Alfonso d’Este, Rome, April 7, 1520) (*10*). Whatever the cause of the artist’s death, the profound impact from his work remains for generations. As attributed to German Nazarene painter Johann Friedrich Overbeck, who was among those present in the Pantheon for the opening of Raphael’s tomb, “Alas, the spirit of the great artist remains buried deeper than his bones.”
